# A Case of Prepubertal Ovarian Tissue Cryopreservation in Metachronous Bilateral Mature Ovarian Teratoma Requiring Bilateral Oophorectomy

**DOI:** 10.1055/a-1926-2053

**Published:** 2022-11-11

**Authors:** Tom Malik, Robert Wheeler, Nigel J. Hall, Juliet Gray

**Affiliations:** 1 Department of Paediatric Surgery and Urology, Southampton Children's Hospital, Southampton, United Kingdom; 2 Department of Paediatric Surgery, Southampton Children's Hospital, Southampton, United Kingdom; 3 Faculty of Medicine, University of Southampton, Southampton, United Kingdom; 4 Centre for Cancer Immunology, Faculty of Medicine, University of Southampton, Southampton, United Kingdom

**Keywords:** mature teratoma, dermoid cyst, ovarian tumor, ovarian tissue cryopreservation, fertility

## Abstract

Mature ovarian teratoma has the potential to occur metachronously in the contralateral ovary. There are significant implications for fertility as bilateral oophorectomy may be indicated. In prepubertal girls, ovarian tissue cryopreservation (OTC) offers the only possibility of a future biological pregnancy but outcome data are limited. We present a case of prepubertal OTC in a 12-year-old girl undergoing a second oophorectomy for metachronous contralateral mature teratoma. We offer a discussion of the challenges that emerged regarding perioperative decision-making, balancing the need for safe oncological resection with the desire to preserve fertility.

## Introduction

Mature teratoma is the most common benign ovarian tumor affecting children. 1 Arising in multiple germinal layers, mature ovarian teratomas are characterized by the presence of ectopic tissue and display a preponderance for ectodermal proliferation. 2 Treatment requires surgical excision, either ovarian sparing or by oophorectomy. 3 Though complete resection is associated with a good prognosis there remains a risk of metachronous contralateral disease, the magnitude of which is uncertain (reported at 4–23% in several European studies 4 5 6 ). Girls with mature ovarian teratoma in the United Kingdom undergo regular postoperative surveillance with ultrasound scan to identify contralateral metachronous disease. 3 If this occurs then infertility is an inevitable sequel when bilateral oophorectomy is required. While ovarian-sparing surgery would likely preserve fertility in these rare cases, it may not be possible. This could be due to concern of a malignant tumor prior to excision, prompting oophorectomy as a safe oncological procedure, or due to it being impossible to identify any macroscopically normal ovarian tissue at the time of surgery. Indeed, oophorectomy is the recommended treatment for any ovarian mass for which the diagnosis is uncertain. 7

For children with cancer, the U.K. National Institute for Health and Care Excellence advises considering options for fertility preservation before treatment is started. 8 The only method available for prepubertal girls is ovarian tissue cryopreservation (OTC). 9 This involves retrieval of ovarian tissue (by ovarian biopsy or oophorectomy) which is then frozen. If the patient is rendered infertile by their oncological disease or its treatment (chemo-/radiotherapy, bone marrow transplant, or surgery) and later wishes to attempt a biological pregnancy, the tissue can be thawed and autotransplanted into the ovarian medulla or an adjacent peritoneal window.

Evidence concerning the efficacy and morbidity of OTC in prepubertal girls is limited. For girls with certain types of cancer and those undergoing bilateral oophorectomy for other reasons, however, it offers the only hope of fertility preservation. We present a case of oophorectomy and OTC for the treatment of metachronous bilateral mature ovarian teratoma in a prepubertal girl. The challenges which have emerged following analysis of the case are discussed.

## Case Report

**Fig. 1:**
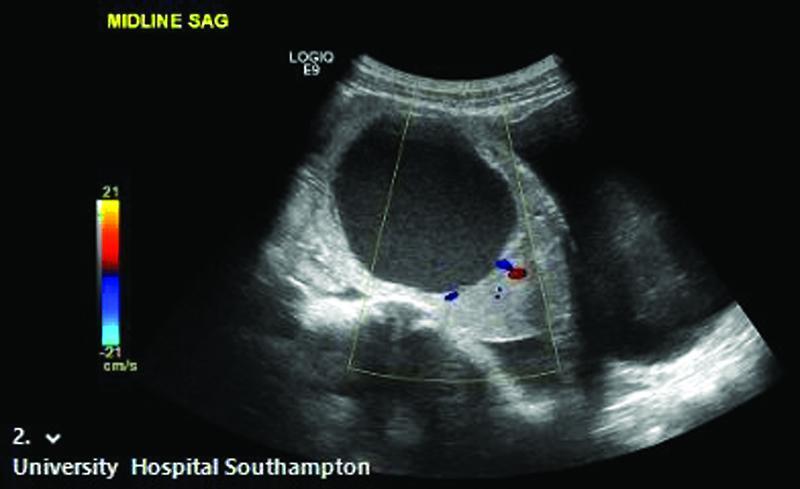
Ultrasonography demonstrating a sagittal view of suspected left adnexal mass. Laparoscopy confirmed that the tumor was in fact associated with the right ovary.

A premenarchal 11-year-old girl presenting with abdominal pain was found to have a left iliac fossa mass. Ultrasonography demonstrated a 7.3-cm left adnexal mass with a central cystic component and peripheral rim of soft tissue ( Fig. 1 ), associated with a small volume of free fluid in the pelvis. Alpha-fetoprotein, human chorionic gonadotropin, CA-125, and lactate dehydrogenase were normal.

**Fig. 2:**
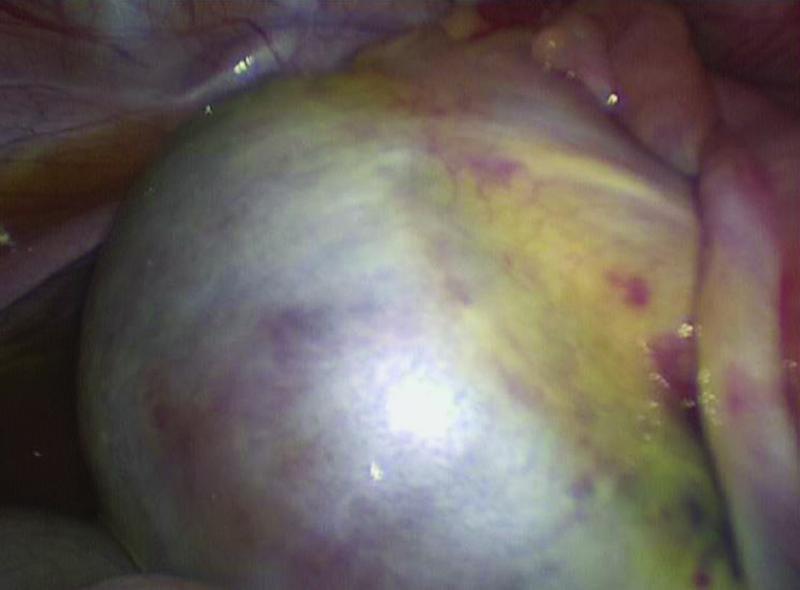
Laparoscopic view of the right-sided mature ovarian teratoma.

Contrary to preoperative clinical and radiological findings, laparoscopy identified a right ovarian mass comprising cystic and solid components with no normal ovarian tissue visible ( Fig. 2 ). The reason for discrepancy between pre- and intraoperative findings was not clear, other than that the anatomy was distorted by the presence of the large mass. Inspection of the left ovary, left fallopian tube, and uterus revealed no abnormality and no other intra-abdominal pathology was evident. The mass was delivered through a muscle-sparing Pfannenstiel incision and excised, with presumed en masse resection of the right ovary. Histopathology demonstrated a completely excised mature ovarian teratoma with no malignant features. There was no normal ovarian tissue visible on microscopy, save for a small amount of hemorrhagic and edematous ovarian stroma. Given the risk of metachronous disease, annual follow-up was arranged with pelvic ultrasonography at each appointment.

**Fig. 3:**
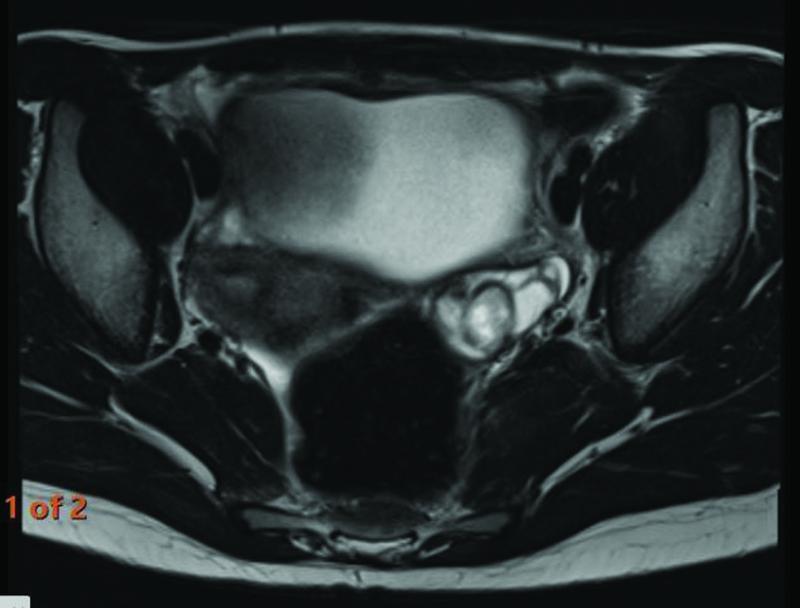
Magnetic resonance imaging (MRI) demonstrating an axial view of the metachronous left ovarian teratoma preoperatively.

**Fig. 4:**
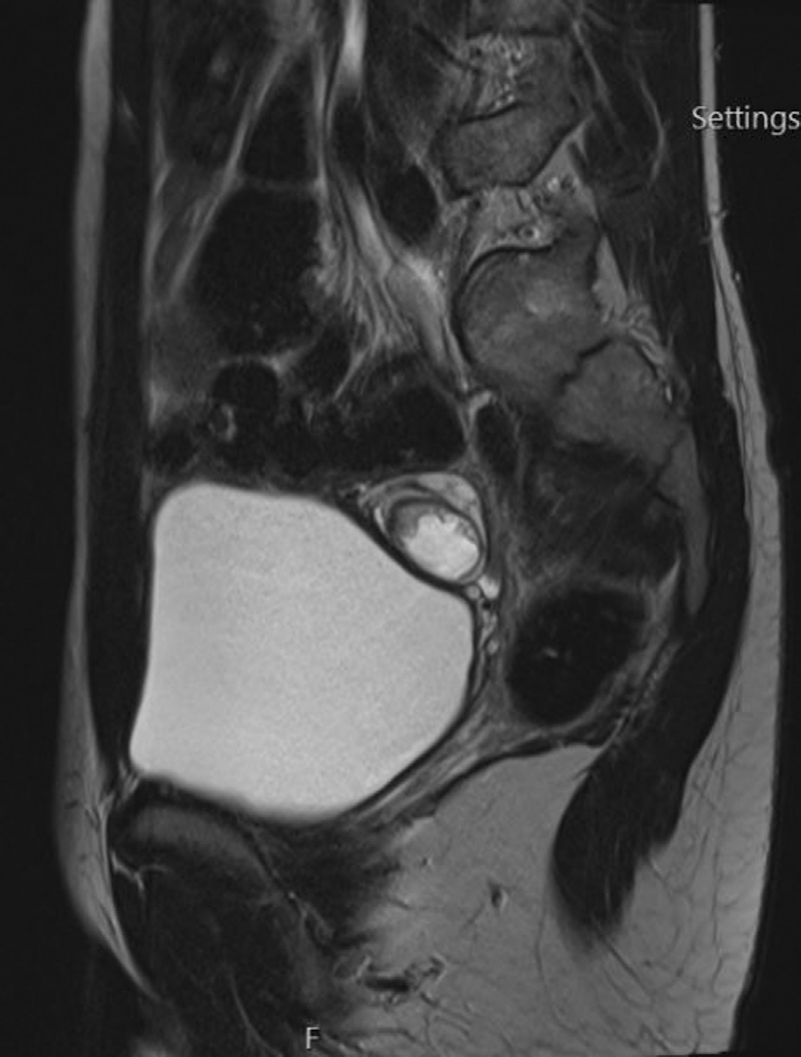
Magnetic resonance imaging (MRI) demonstrating a sagittal view of the metachronous left ovarian teratoma preoperatively.

At follow-up 1 year after surgery, routine ultrasound revealed a 1.7-cm area of increased echogenicity concerning for calcification in the left ovary. Magnetic resonance imaging demonstrated a multicystic left adnexal lesion containing fat ( Figs. 3 and 4 ). Tumor markers were again normal. The patient received multidisciplinary input from consultants in pediatric oncology, pediatric surgery, and reproductive medicine alongside a fertility counselor. A decision was made to attempt ovarian-sparing excision of the tumor to preserve fertility. It was, however, agreed preoperatively that OTC would be performed if oophorectomy became necessary.

At laparoscopy, the left ovary appeared pathological but intra-abdominal inspection was otherwise normal. The previous Pfannenstiel incision was reopened and the left adnexal structures were delivered through the wound. The tumor's limits were difficult to discern, with no macroscopically normal ovarian tissue visible, therefore oophorectomy was performed. Following resection, the specimen was dissected ex vivo and a sample of apparently normal ovarian tissue was sent for cryopreservation. Histopathology demonstrated a mature ovarian teratoma with clear margins and no malignant characteristics.

Multidisciplinary care continued postoperatively. There were no oncological or surgical concerns and hormone replacement therapy was commenced. The patient received counseling and was reviewed by a dietitian as her weight remained static during a 6-month period postsurgery. She continues to be followed up by a general pediatrician and pediatric endocrinologist. Her frozen sample of ovarian tissue is now stored securely and will be made available if she wishes to attempt pregnancy at a later date.

## Discussion

We have described a case of bilateral metachronous oophorectomy and OTC to treat metachronous bilateral mature ovarian teratoma in a prepubertal girl. This is a rare condition, but has been reported in up to 23% of girls with mature ovarian teratoma. 4 5 6 The case highlights important challenges for those caring for girls with this pathology.

It is believed that the ideal method of fertility preservation in children with mature ovarian teratoma is to perform ovarian-sparing tumor excision to preserve healthy ovarian tissue. The desire to preserve fertility must, however, be balanced against the need to perform an oncologically safe resection. Adherence to the principles of oncological surgery entails complete tumor dissection, staging, and avoiding tumor spillage. 10 In cases of suspected mature ovarian teratoma, the surgeon must still respect these principles as the true nature of the disease cannot be known until histopathological examination has been performed. In their series of children with mature ovarian teratoma, Chabaud-Williamson et al demonstrated complete resection in all cases of ovarian-sparing surgery ( *n* = 10). 5 They recommended that this technique be reserved for tumors suspected to be localized mature ovarian teratoma. This view is supported by recent guidance from the Children's Cancer and Leukaemia Group, which states that an attempt at ovarian-sparing resection is acceptable if mature teratoma is strongly suspected. 3 In addition, a clear plane of dissection between tumor and normal ovary must be visible intraoperatively. 11 In this case, ovarian-sparing surgery was not possible for either side. This resulted in the unfortunate position of a girl rendered infertile following surgical treatment of what ultimately was found to be benign disease.

For situations such as this, OTC represents the only option for a future biological pregnancy. However, the sparsity of evidence concerning the efficacy and safety of prepubertal OTC presents a challenge to those considering its undertaking. Limited reports exist regarding the efficacy of OTC when tissue has been harvested prepubertally, although this is a rapidly developing field. Two cases of successful pregnancy have been reported following prepubertal OTC 12 13 and induction of puberty has been reported following autotransplantation of prepubertally cryopreserved ovarian tissue. 14 15 While generally a safe procedure, harvesting of ovarian tissue may require additional surgery with the inherent associated risks. Due consideration of these risks should be made in particular in cases where laparoscopy would otherwise not be required and the risk of gonadal failure (usually related to treatment of oncological or hematological disease) may be difficult to quantify. 16 There is currently no standardization of service provision for OTC in prepubertal girls in the U.K. At our center, it is considered in individual cases at high risk of infertility secondary to treatment for benign or malignant disease.

In conclusion, the possibility of metachronous contralateral disease necessitating bilateral oophorectomy should be considered in all children with mature ovarian teratoma. Ovarian-sparing surgery should be considered at index operation, and regular ultrasound surveillance should be undertaken. The efficacy of prepubertal OTC remains uncertain and it is important to engage full multidisciplinary team discussion prior to its undertaking. Informed consent regarding resectional strategy and OTC mandates full disclosure of the associated benefits and risks, guided by the best available evidence.

## Notes

New Insights and the Importance for the Pediatric Surgeon The potential for metachronous contralateral should be considered at index operation and a decision made regarding resectional strategy. Ovarian tissue cryopreservation should be discussed in a multidisciplinary setting and with careful consideration of the risks and benefits guided by best available evidence.
